# A Programmable Readout Pixel Image Sensor

**DOI:** 10.3390/s26165245

**Published:** 2026-08-19

**Authors:** Joseph P. Lazzaro, Karthik R. Venkatesan, Eric R. Fossum

**Affiliations:** Thayer School of Engineering at Dartmouth, Dartmouth College, Hanover, NH 03755, USA; karthik.raj.venkatesan.th@dartmouth.edu (K.R.V.); eric.r.fossum@dartmouth.edu (E.R.F.)

**Keywords:** CMOS image sensor, charge-coupled device, CCD-in-CMOS, Skipper-in-CMOS, deep sub-electron read noise

## Abstract

In this paper, a pixel architecture that can be re-programmed to favor low noise, high speed, high dynamic range, or other characteristics desired in scientific imaging is proposed. Termed a programmable readout (PRO) pixel, this novel design combines a pinned photodiode, a deep-buried-channel CCD router, and multiple in-pixel amplifiers of different characteristics. By clocking the CCD router with different sequences, photogenerated charge can be steered to an appropriate readout amplifier given the application. The CCD router can be further programmed to store charge from multiple frames. The concept of such a pixel and its operation are described in detail and TCAD simulations aid in this discussion. To demonstrate this concept of programmability, a test chip is made with a CCD router and two in-pixel amplifiers: a floating diffusion amplifier (FDA) for fast readout and high-illumination imaging and a floating gate amplifier (FGA) for a low-noise Skipper-in-CMOS readout for low-light imaging. The test chip contains a 36 × 94 array of 20 µm pixels and contains 9 different pixel variants for experimental analysis. At the end of the paper, preliminary images captured from the different amplifiers are presented which demonstrate the ability to move charge to the selected readout while showcasing the different output characteristics of the two readout amplifiers. Furthermore, the output image from the Skipper-in-CMOS is compared across different numbers of non-destructive samples to demonstrate its noise-reduction capability.

## 1. Introduction

State-of-the-Art image sensor research seeks to improve performance characteristics such as noise, frame rate and dynamic range. Certain applications, including security surveillance, machine vision, microscopy, and other scientific applications, often require some combination of low-noise, high-frame-rate and high-dynamic-range capabilities simultaneously. However, optimizing a pixel design for one of these desired characteristics typically requires tradeoffs with the others. Because of this, the specifications of a pixel are usually carefully chosen for a specific application and fixed early in the design phase with limited flexibility to change performance post fabrication.

Low-noise CMOS image sensors have achieved deep sub-electron read noise (DSERN; e.g., less than 0.30 e^−^ rms) through the use of low-capacitance sense nodes and signal processing but require advanced CMOS image sensor fabrication nodes that are not easily accessible for low-volume applications [1]. While readout of CMOS image sensors is typically performed using the widely adopted, destructive floating diffusion amplifier (FDA) readout developed for charge-coupled devices (CCDs), some recent research efforts in low-noise readout have focused on floating gate amplifiers (FGA) as sense nodes in solid-state image sensor pixels due to their non-destructive sensing capability. FD readouts allow only a single correlated double sampling (CDS) cycle, although the signal and reset from each cycle can be sampled multiple times using correlated multiple sampling (CMS), which affords some noise reduction beyond CDS. By contrast, FG readout can be used to improve signal-to-noise ratio (SNR) by re-sampling of the photoelectrons using CDS and multiple readout cycles. Furthermore, these devices can be fabricated in a sunset-foundry node, such as 180 nm, that are more accessible and affordable.

The concept of using a floating gate sense node with multiple samples to reduce noise was proposed by Wen at Fairchild in 1975 for CCDs [2], adapted for use in scientific CCDs by Chandler et al. at Ford Aerospace and the NASA Jet Propulsion Laboratory (JPL) in 1990 as a “Skipper CCD” [3] and subsequently proposed for use in a CMOS image sensor in 1993 at JPL by Fossum [4]. The SNR improvement by re-sampling has garnered renewed interest recently due to the low-noise requirements needed by scientific detectors for direct detection of dark matter [5] and the discovery of habitable worlds around other stars [6]. Implemented in CCD technology and in CMOS (“Skipper-in-CMOS”) [7,8], DSERN via charge skipping has been demonstrated, albeit with hundreds or thousands of re-sampling cycles and with operation at cryogenic temperatures to suppress dark current given the long integration and readout times associated with these applications. It is noted that with DSERN, not only can single photoelectrons be discerned with high confidence, but photoelectron number can also be determined with high confidence, even at numbers in the hundreds [9].

The floating gate re-sampling technique achieves low effective read noise by moving a collected charge packet under a floating gate where the induced voltage change on the floating gate is sampled. The charge is subsequently moved away to a storage area, restoring the initial potential in the sense node while preserving the charge packet. Once the empty well potential is read out for CDS, this charge is moved back to the floating gate and sampled again. After many reads, these samples are averaged, and the SNR is improved by the square root of the number of samples. As was mentioned, the low-noise performance of a re-sampling device comes at the cost of a greatly increased time per readout due to many readout cycles, limiting its use to scientific, still-frame imaging where fast acquisition of an image is not a requirement. For example, in [7] an effective read noise of 0.15 e^−^ rms was achieved after 3025 readout cycles at 2 × 4.66 μsec/cycle or 28.2 msec per pixel at a temperature of 118 K. For a 1 Mpix sensor, that would correspond to almost 8 h to read out one image through a single readout amplifier. In contrast, the goal in this work is to achieve DSERN performance in less than 50 readout cycles at room temperature. Additional goals include high frame rate and high dynamic range.

High-frame-rate CIS pixel design typically involves careful optimization of pinned photodiode (PPD) geometries to force a gradient potential towards the edge of a transfer gate such that electrons do not need to diffuse from one edge of the PPD to the other and can move predominantly via drift which is known to speed up the charge transfer process [10,11]. In modern 4T pixel design, this charge still needs to be transferred to a floating diffusion for readout, a process which adds time to the readout of a pixel. Efforts have been placed on optimizing this transfer process as well, such as the use of charge-sweep gates [12] which establish a stronger electric field pointing toward the sense node compared with a single transfer gate. In addition to charge transfer, the well-established process of resetting the sense node and performing correlated double sampling adds time to the readout process as this is another signal that must settle, be sampled, and be processed.

There are many known techniques to achieve high dynamic range, such as multiple exposure with different integration times [13], logarithmic response pixels [14], multiple in-pixel gain [15], and dual gain column amplifiers [16]; however, recent attention has shifted toward cascaded integration [17], now called lateral overflow integration capacitor (LOFIC) pixels for PPD detectors [18] as a solution to capture dim and bright scenes in a single frame [19]. Here, collected charge that exceeds the full well capacity (FWC) of the PPD spills over to the FD and then to one or more storage capacitors. For low signal levels, the charges are contained within the PPD and the pixel can be operated as a 4T pixel. For large signal levels, the FD and storage capacitor(s) accumulate the overflow charge which can be read out through the source follower. With this technique, dynamic range is extended but predetermined in the design phase. The readout technique proposed in this paper provides additional flexibility between the low- and high-dynamic-range modes of operation.

Pixels utilizing multiple transfer gates, or taps, from the PPD to different storage or sense nodes have also been recently proposed to improve dynamic range [20,21]. These involve programmatically steering the charges to one or more readouts with the same or different conversion gains to adjust the effective exposure time. However, these techniques still employ a destructive read and the charges can be read only once.

Taking inspiration from the above techniques for low-noise, high-speed, and HDR imaging, as well as recent work implementing CCD-in-CMOS [22,23], this paper proposes a novel technique to enable all these capabilities in a single pixel. Termed the programmable readout (PRO) pixel, this design allows a user to easily tune the aforementioned tradeoffs at run-time and provides a single sensor solution to a wide range of applications that previously required different, specifically tailored sensors each, in our case, for use in photonic integrated circuit (PIC)-based portable biosensors and in the imaging of Cherenkov light during medical radiation therapy. Implementation comes at the cost of pixel size and a need for programmable timing and control circuits for the image sensor. For low volume applications, such a trade might be economically attractive. The PRO pixel concept and design are further explained, and simulation and early experimental results are provided in the subsequent sections.

## 2. PRO Pixel Concept

The main concept of the PRO pixel is the ability to programmatically steer and transfer a collected charge packet to one or more in-pixel storage sites and readout amplifiers of different characteristics, such as low noise or high dynamic range, within the pixel (or shared with adjacent pixels) using well-known charge transfer device technology.

**Figure 1:**
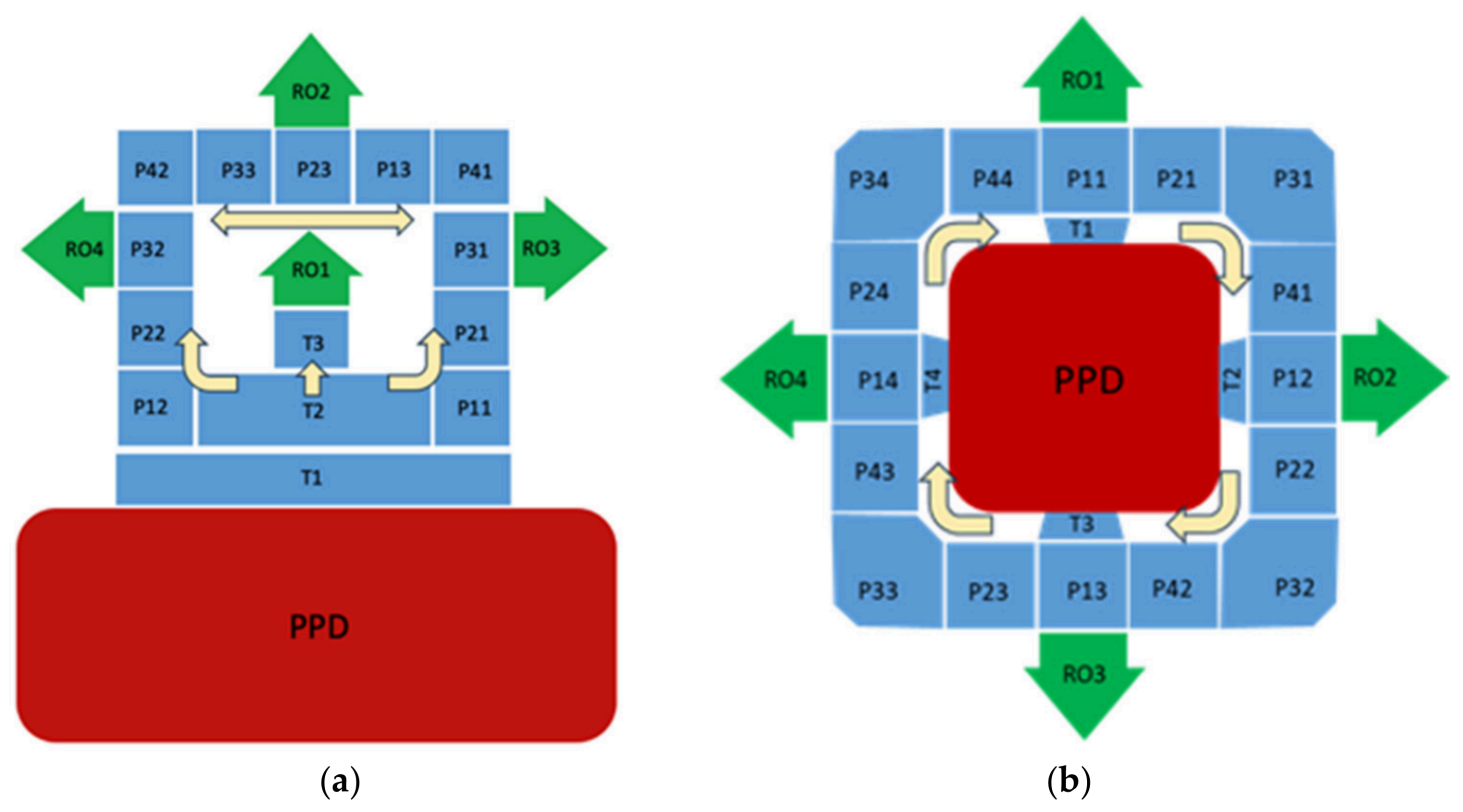
(**a**) PRO pixel concept with an adjacent PPD. (**b**) PRO pixel concept with a centered PPD. Both designs allow for readout by four different amplifiers of different characteristics.

An example of this concept is shown in Figure 1a where photogenerated carriers collected in the PPD are moved through a transfer gate (T1), indicated by the beige arrows, into a CCD router (i.e., P1n, P2n, etc.) that leads to different output amplifiers, shown in green (ROn). Figure 1b shows another conceptual illustration of the many possible configurations of a PRO pixel.

Both the transfer gates and the router comprise closely spaced polysilicon gates over a deep buried channel. By clocking these gates, charge can be transferred from one phase to another [24]. As an example in Figure 1a with appropriate biases, the charges collected in the PPD can either be moved to RO4 through T2, P12, P22, P32 or to R03 through T2, P11, P21, P31. An important attribute of the PRO pixel design is its ability to move charge bidirectionally in the router which enables the implementation of a multiple-sampling FG readout.

By placing a deep buried channel beneath the transfer and router gates, a charge packet moving along the CCD will travel beneath the Si surface as long as an appropriate bias voltage is selected. This reduces carrier interactions with interface traps at the Si-SiO_2_ interface and thus allows for more efficient charge transfer and lower dark signal (and noise) performance. In order to keep these carriers below the surface, it is important to operate the router gates in depletion so as to not pull the charge out of the buried channel and up towards the gate [25]. Careful selection of the buried channel implantation dose also helps to keep charge away from the surface by creating a deeper potential well in the bulk than this interface, even when a bias is applied.

With the ability to bring a charge packet to multiple different readout stages, the PRO pixel can support a wide variety of imaging modes. For example in Figure 1b, if each of the four readout stages comprise floating gate sense nodes, a collected charge packet may be continuously moved in a clockwise or counter-clockwise direction and be sampled non-destructively as it passes through P11, P12, P13, and P14. Similar to the aforementioned Skipper-in-CMOS, this would allow the averaging of these reads to reduce the read noise to deep sub-electron levels with enough laps around the device. If these readout nodes are all constructed the same, one lap around the pixel would result in four samples that could be averaged. Conversely, if they have different characteristics, such as a different conversion gain for example, the same charge packet could be read four different ways in one lap and not be limited to the CG of one single device, allowing both low light and bright scenes to be captured. If higher frame rate is required, a readout amplifier with desired characteristics can be chosen and a direct path to that sensing node can be taken (e.g., T4 → P14 → R04 for a low conversion gain, or T2 → P12 → RO2 for a high conversion gain in Figure 1b). In Figure 1a, gates T1, T2 and T3 can be designed to operate like the charge-sweep gates described earlier and can be used for ultra-fast imaging. It is easy to see that there are many design possibilities for a PRO pixel.

While the clocked phases primarily serve to move charge to a desired location within the device, they also play another integral role as charge storage regions. By holding a constant bias over one phase and a lesser (assuming an n-type buried channel) bias on its adjacent phases, the charge packet will remain stationary and preserved under that phase’s gate, as long as the dark current is insignificant. While the full well capacity of the phase limits the amount of charge which can be stored under it at a time, biasing the adjacent phase to the same level now doubles this full well, and thus the amount of charge stored can be programmed by the amount of phases used as this storage region. This then opens the door to HDR imaging, ToF imaging, FLIM imaging, etc.

It should be noted that while the shape of the PRO pixel charge router and the number/characteristics of each readout amplifier are predetermined in the design phase, the way in which the biases and timing are applied to the gates is not. By reprogramming the control signals which apply the biases, one single PRO pixel can perform many different tasks.

## 3. Test Pixel Design and Operation

**Figure 2:**
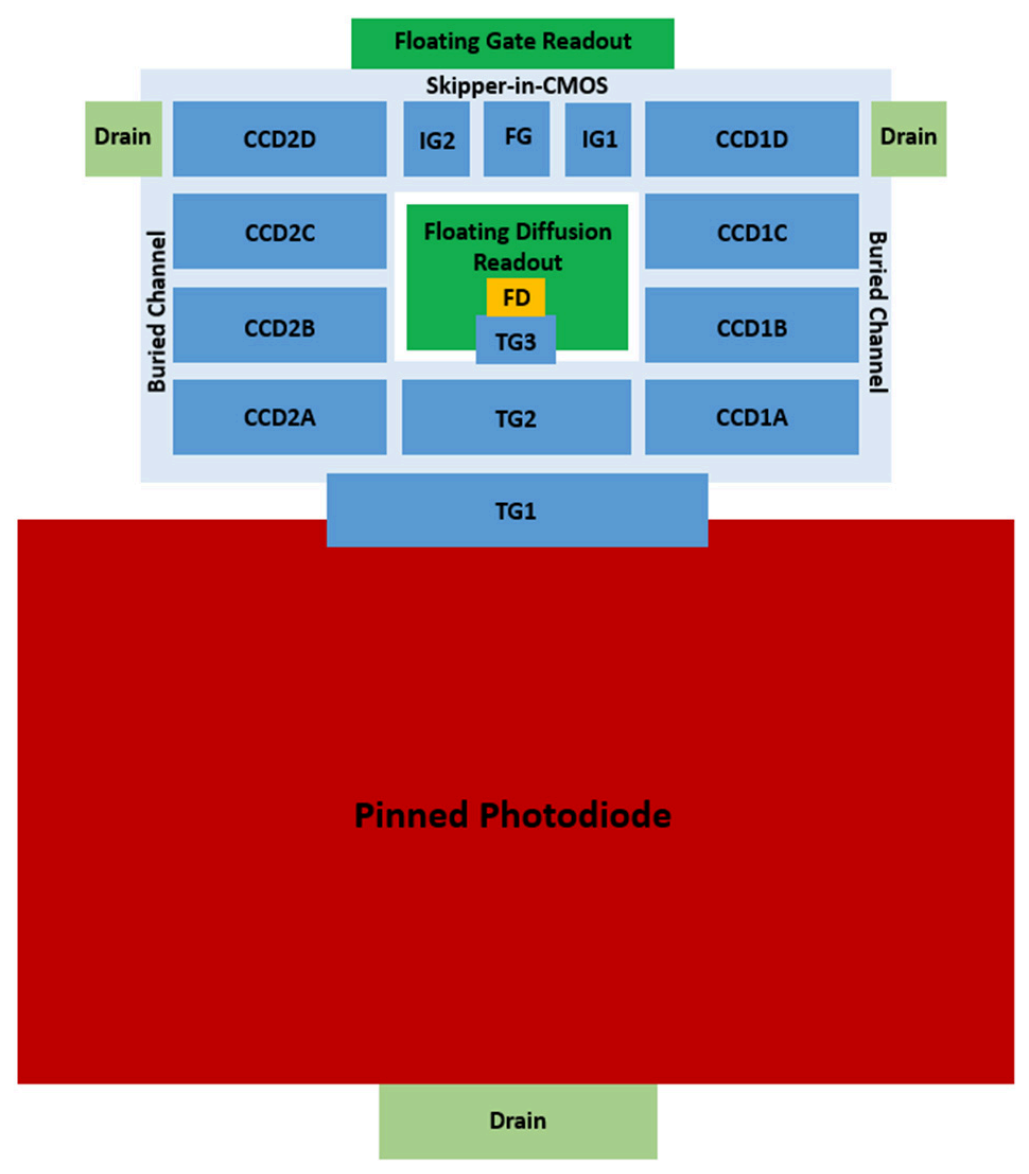
Test chip 20 × 20 μm PRO pixel showing simplified plan-view layout.

A test pixel was designed for the X-FAB (Erfurt, Germany) XS018 process including CCD-in-CMOS, as illustrated in Figure 2, based on the adjacent PPD PRO pixel concept from Figure 1a. Here, two different readout amplifiers exist: one floating diffusion and one non-destructive floating gate, each with its own source follower. Three gates of decreasing widths and lengths, TG1 TG2 and TG3, separate the PPD from the floating diffusion to function as charge-sweep gates for ultra-fast charge transfer and readout if desired by a user. Two 4-phase CCDs, named CCD1 and CCD2 with phases labeled in alphabetical order, interface with TG2. These CCDs, along with TG2, play the role of the ports in Figure 1a. At the end of each CCD is an FG readout to implement the Skipper-in-CMOS. In this Skipper-in-CMOS, CCD1D or CCD2D act as storage gates to hold a charge packet while the reset level is being sampled under the floating gate, FG. Two isolation gates, IG1 and IG2, separate the Skipper storage gates from the floating gate. These gates go low during reset sampling so as to not cause a fringing field bias on the floating gate which would prevent an accurate reset reading. When moving charge to the floating gate to sample the signal, the isolation gates are pulsed with a bias greater than that on the storage gate but less than the potential on the floating gate, set by a reset transistor. Both the CCDs and the PPD have a highly doped and positively biased gated charge drain region. This is essential for fully clearing charge stored in the CCDs for a new frame as well as for allowing global shutter operation and anti-blooming. Not shown in Figure 2 but also essential to proper operation of a PRO pixel is light shielding over the phases and transfer gates to ensure no electrons generated outside of integration enter the storage regions.

In this design, the CCD gates are designed based on the maximum amount of charge that needs to be stored in any given frame. The spacing between gates is the minimum allowed by the fabrication process. Closely spaced gates are integral to a CCD-in-CMOS device to avoid formation of a potential barrier between the gates, which could result in inefficient charge transfer despite being properly biased for transport. Even with the buried channel, it will be difficult to move charge around a PRO pixel if the fringing field from neighboring gates does not fully remove this barrier.

The Skipper-in-CMOS gates are noticeably smaller than the CCD gates. When using an FG readout, a tradeoff exists between the FWC of the floating gate and the noise performance of the pixel. A larger floating gate can hold more of the signal charge before saturation; however, this comes at a cost of larger capacitance on the sense node and thus a reduction in conversion gain. Since the effective read noise achieved using a number of readout cycles, N, is the noise of a single readout cycle divided by the square root of N, the most efficient way to reduce the number of readout cycles required to achieve a noise specification, and thus the time required to read out a pixel, is to use a large conversion gain and reduce the noise of a single read. As such, the Skipper-in-CMOS gate dimensions were chosen to balance this tradeoff, with an approximate FWC of 3700 e^−^ and the goal of achieving less than 0.5 e^−^ rms input-referred read noise within 50 readout cycles.

The design of the source follower, which converts the signal charge in the sense node to a proportional voltage, also requires careful optimization to achieve desired noise performance. Here, a tradeoff exists between conversion gain and 1/f noise. A smaller gate reduces capacitance on the sense node allowing for higher conversion gain, but it has been widely observed and reported that a smaller source follower channel results in higher 1/f noise [26]. To analyze this tradeoff, several different source follower variants have been implemented. These variants include three NMOS devices of varying gate lengths ranging from 350 nm to 800 nm, a buried-channel NMOS with a gate length of 650 nm, and four multi-gate source followers (MGSF) inspired by the work in [26]. The MGSF seeks to circumvent the tradeoff between conversion gain and 1/f noise through the use of an additional gate, termed the guard gate, which adds additional channel length without affecting the sense node capacitance. With this, the source follower input gate, termed the modulation gate, can be made very small without increasing 1/f noise. Both surface-channel and buried-channel MGSFs are included for performance comparison. Table 1 summarizes the different transistors and their dimensions in these experiments.

Additionally, two experimental PPD structures were designed to analyze the tradeoff between dark current and fill factor. In one variant, the PPD edges are slanted to join the corners of the buried channel of the CCD. The back half of this PPD also does not contain sharp edges to lower the dark current in this device. The second PPD, as depicted in Figure 2, is rectangular with a length of 7.815 µm and a width of 18.355 µm to maximize fill factor and FWC. Here, the PPD extends 3.33 µm beyond the edge of the buried channel on both sides. Given the number of control signals and gates in a PRO pixel, low fill factor, or conversely large pixel pitch, is the main design shortcoming, especially in a 180 nm process. In this 20 µm pitch pixel, a fill factor of 33% is achieved, which is more than acceptable for the initial target application. Microlenses or backside illumination could enhance the effective fill factor, but neither capability was included in this initial test design.

**Figure 3:**
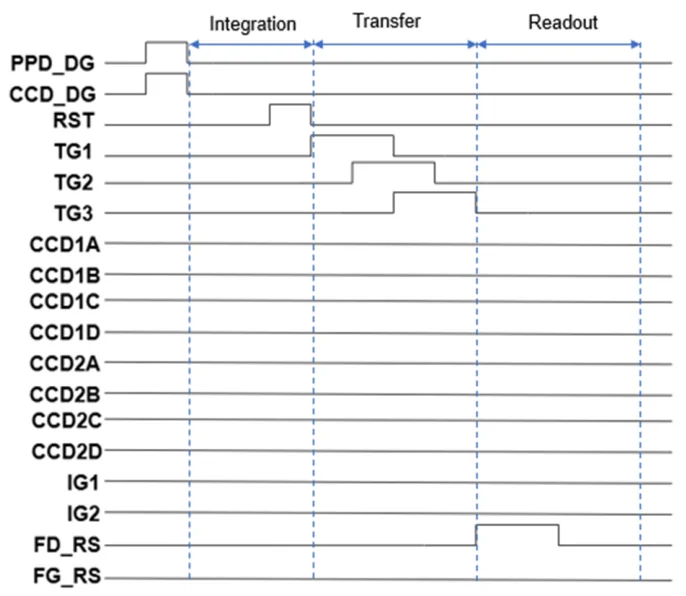
High-frame-rate readout timing diagram.

The major advantage of the PRO pixel is the ability to configure its timing, during operation, to prioritize certain performance parameters over others. For example, the readout mode involving the fewest number of control signals, and thus the simplest way to operate the pixel, is a high-frame-rate floating diffusion readout. A timing diagram for this mode is provided in Figure 3. Here, the three transfer gates are pulsed in a charge-sweep fashion, but no storage gates or Skipper-in-CMOS gates are utilized and remain low for the entire operation.

**Figure 4:**
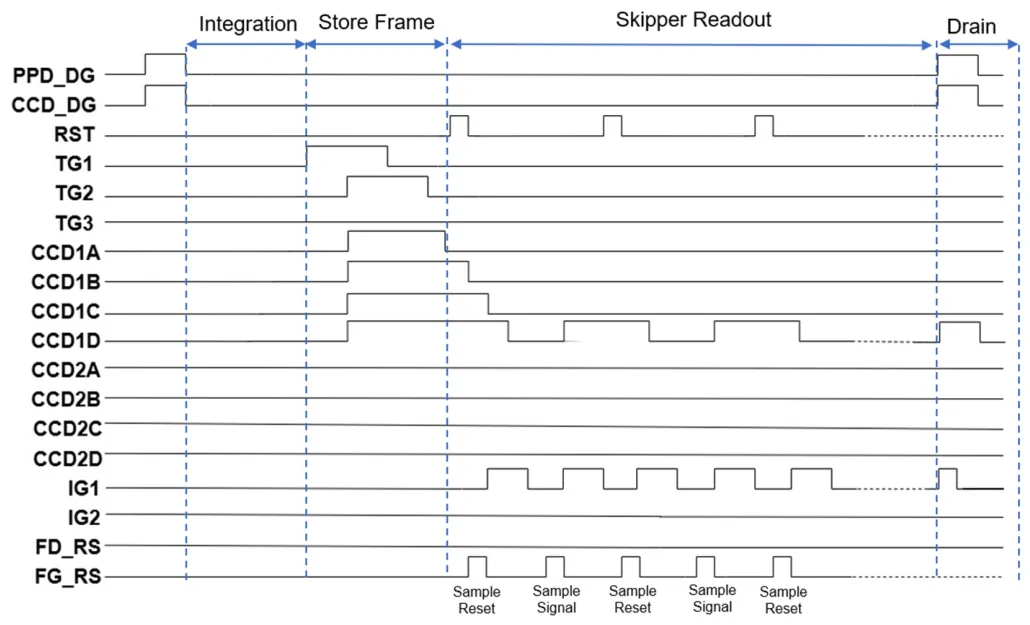
Single-frame, low-light Skipper-in-CMOS readout timing diagram.

A single-frame, low-light Skipper-in-CMOS readout requires more control signals to be programmed, as shown in the timing diagram of Figure 4. Here, charge is moved via TG1 and TG2 from the PPD to CCD1. The stored charge spread out amongst CCD1 is all moved to CCD1D to begin the skipping process. CCD1D and IG1 work together to move charge between CCD1D and the floating gate, allowing repeated, non-destructive read of the reset level and signal level through the floating gate source follower. In this readout mode, the reset is pulsed each time that the charge packet is sampled and moved back to CCD1D, but this may not be necessary: once the charge is moved away from the FG, the potential on the sense node should return to the initial reset level at the start of the frame. The number of read cycles can be programmed as desired given the noise and frame rate specification of the application.

**Figure 5:**
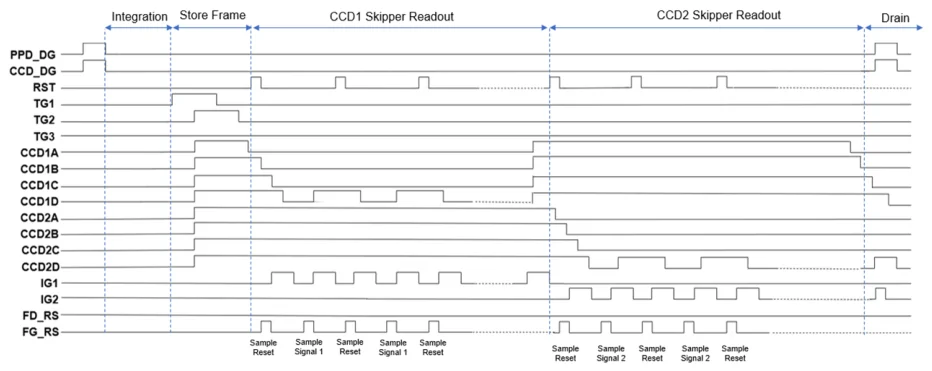
High-FWC Skipper-in-CMOS readout timing diagram.

A high-dynamic-range readout mode can be realized by utilizing the charge storage capacity of both CCD ports. As shown in Figure 5, this operation works by collecting up to double the FWC of the floating gate in the PPD, turning on the transfer gates, and biasing both CCDs high. If constructed and biased the same, the collected charge will be equally split amongst these two CCDs. Charge from one CCD is then moved to the Skipper-in-CMOS output stage to begin the multiple-sampling process, while the other CCD remains in storage mode. After the first half-packet has been sampled sufficiently, this charge is moved back into storage and the second half-packet is moved to the Skipper and sampled. Finally, all charge is dumped through the drain gates, and the low noise reading of both half-packets are summed in post-processing to form one read. This charge splitting concept could also be applied to a floating diffusion readout to achieve high dynamic range.

These three examples show only a portion of the different ways the designed PRO pixel can be operated. It can be easily imagined from these examples how ToF, FLIM, or other multiple-frame imaging techniques may also be performed with these storage gates and different readout amplifiers. Following characterization and validation of this test pixel, a more general PRO pixel with additional readout amplifiers will be designed and fabricated.

## 4. Modeling Results

**Figure 6:**
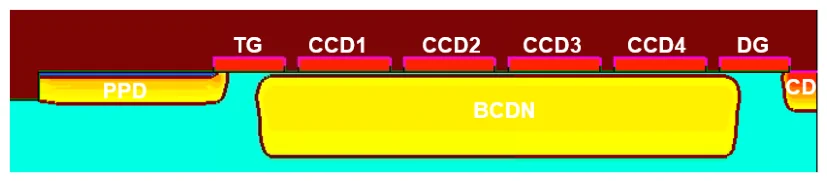
Sentaurus TCAD process model used to simulate the CCD-in-CMOS in the PRO pixel, where the BCDN has a lower implantation dose but larger junction depth compared with the PPD.

While designing this pixel, TCAD (Sentaurus 2016.03) was employed to model and simulate charge transfer throughout the device. It is noted that due to the proprietary nature of the foundry process, they could not provide full process details, so in this work we used our best estimate of process parameters (e.g., implant doses and energies). Figure 6 presents a cross-sectional view of the 2D model, which incorporates all of the components of a PRO pixel aside from the readout amplifiers, which are instead simulated in a SPICE simulator. The modeled components include the PPD, transfer gate, deep buried channel (BCDN), 4-phase CCD (with gates over the BCDN), dump gate (DG), and a highly doped charge drain (CD). The BCDN is modeled with a lower dose of phosphorous (the n-implant) than the PPD but with a higher implementation energy and thus extends deeper into the bulk.

**Figure 7:**
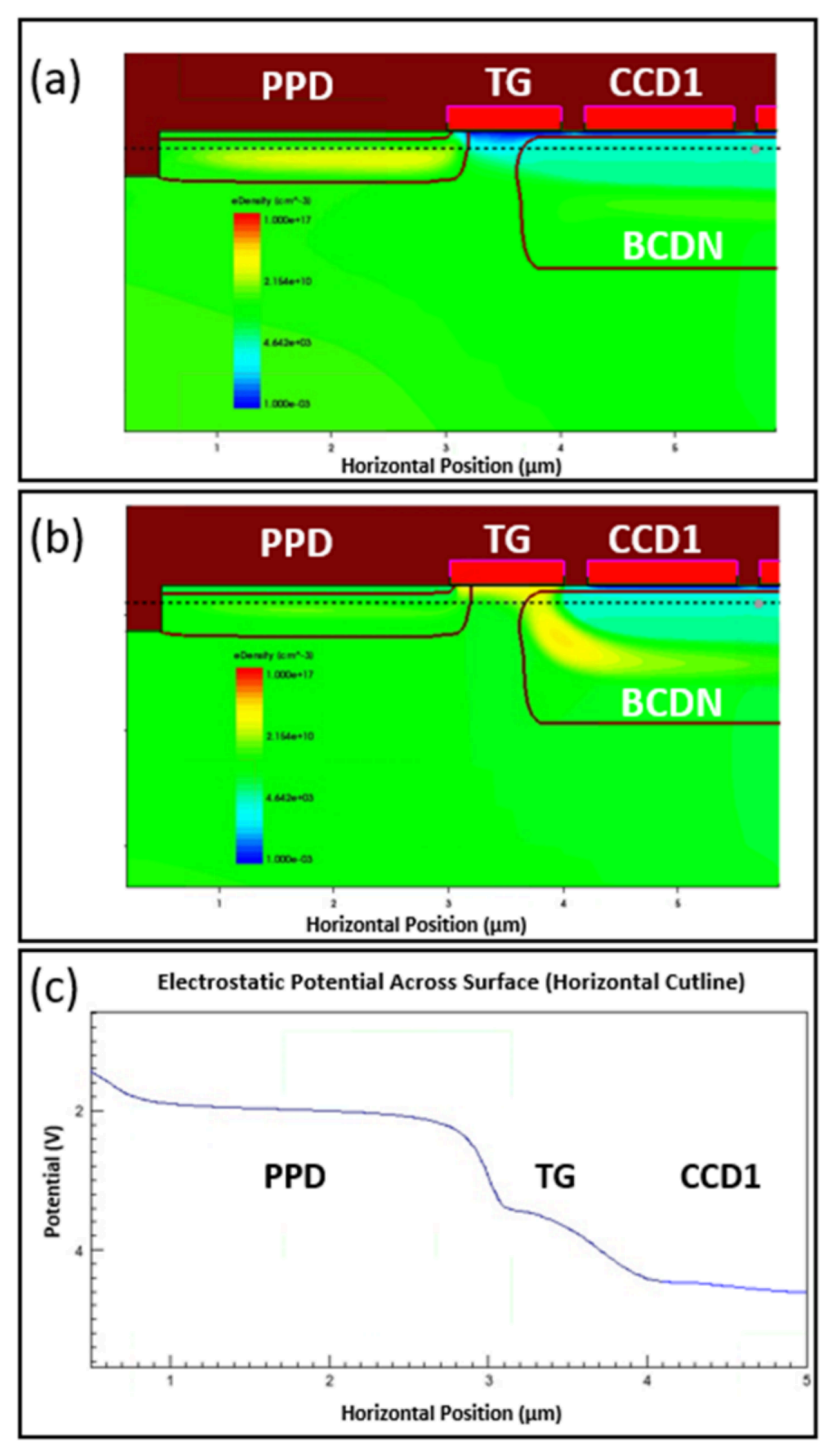
The doping profile of the device and proper biasing of the gates allow for efficient charge transfer from the PPD to the BCDN (**a**,**b**) by creating the potential curve in (**c**) near the surface during transfer.

In a typical 4T pixel, a floating diffusion is placed on the opposite side of the transfer gate with a much higher doping concentration than the PPD. When the transfer gate is pulsed, it provides a deeper potential well than the n-doped PPD, which allows charge to exit the PPD and drift toward the even deeper potential well from the highly doped floating diffusion, allowing for full charge transfer out of the PPD. In the case of the PRO pixel, the potential well in the BCDN is shallower than that of the PPD, meaning that even with the fringing field from the pulsed transfer gate, the charge will not make it over the potential barrier and into the buried channel. To ensure high charge transfer efficiency, the CCD gate adjacent to the transfer gate must also be set to a sufficiently high potential to eliminate this potential barrier. This transfer process was simulated to verify that complete charge transfer from the PPD is achievable with this doping profile while also ensuring proper isolation between the buried channel and the PPD during post-transfer operation. This isolation is essential to avoid charge spillback as well as to not introduce new carriers generated and collected in the PPD after the transfer into the buried channel during storage or readout. Figure 7a,b provide transient snapshots before and during this transfer process, where charge entering the buried channel does so below the surface by biasing the CCD in depletion. Figure 7c shows the potential curve of a horizontal cutline along the surface during this transfer where the barrier between the PPD and BCDN has been effectively suppressed.

**Figure 8:**
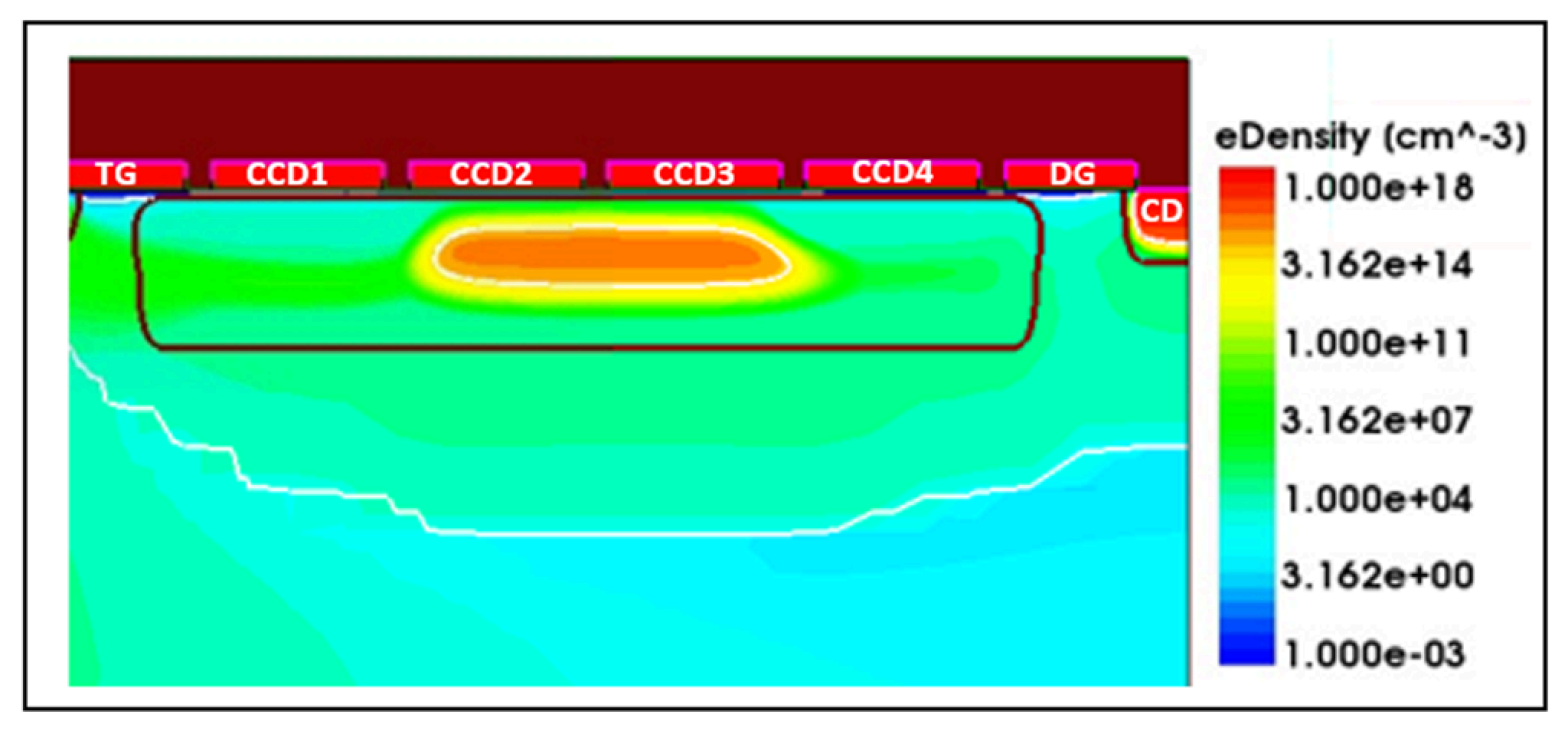
Biasing CCD2 and CCD3 in depletion but higher than CCD1 and CCD4 enables charge storage under these gates and below the surface.

The storage capability of the CCD gates was also simulated and a snapshot of this operation is provided in Figure 8. Again, these gates are biased in depletion to keep the stored charge away from interface states at the surface. In this simulation, more optical generation (1 mW/cm^2^ at 550 nm) was used compared with the simulation depicted in Figure 7 to aid in determining the FWC of these gates. Here, storage is performed with two gates high, but as previously mentioned, any number of gates can be used in this function depending on the amount of charge collected and where it is convenient in the PRO pixel to keep this charge depending on the next readout task.

**Figure 9:**
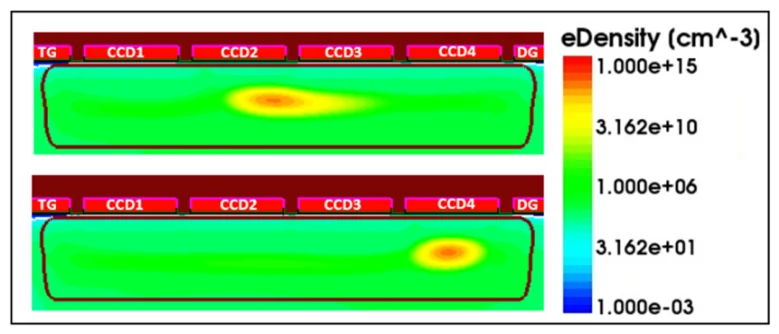
Charge can be moved bidirectionally and below the surface between different gates with proper isolation, allowing for efficient transport to different readout amplifiers and Skipper-in-CMOS operation.

The final operation simulated in TCAD was the Skipper-in-CMOS function of the PRO pixel. Here, a low-light scenario was simulated and it was confirmed that charge can be moved bidirectionally between CCD2 and CCD4 without surface interaction. Sufficient isolation of the charge packet was also observed as shown by the contained charge under CCD4 in the transient snapshot in Figure 9. This isolation is important for accurately measuring both the signal and the reset level without any fringing field effect from CCD3 or charge lag.

The Skipper-in-CMOS simulation also aided in determining the voltage levels required to perform this operation. The four voltage levels used here are provided in Table 2. This result informed the row control circuitry design for the PRO pixel.

In order to achieve the target specification of 0.5 e^−^ rms read noise or less within 50 repeated samples, capacitance extraction of the floating gate sense node layout was performed for five different source follower gate lengths. Flicker and thermal noise contributions were also simulated for each source follower using a SPICE simulator (Cadence SPECTRE 24.1). Table 3 below provides the results of these simulations as well as the expected conversion gain, noise of a single read, and noise after 50 samples of the Skipper-in-CMOS readout. As expected, the 1/f noise decreases and the conversion gain increases as gate length is increased. As noted earlier, NMOS source follower lengths of 350 nm, 650 nm, and 800 nm were included as variants for later experimental comparison of these same metrics.

The above 1/f noise estimates were computed via SPICE simulations. AC noise analysis with BSIM4 models for the source follower devices, provided by the foundry, was performed over a frequency range of 200 kHz to 100 MHz using an industry standard SPICE simulator. Simulation was restricted to a subcircuit comprising the source follower, load current, and passive output loads. The bias point on the source follower gate was set to simulate the reset level used in this design, and the load capacitance was set to the estimated input capacitance of the readout circuit, 1.6 pF. The range of gate lengths in the test chip was chosen to handle the conversion gain versus flicker noise tradeoff given the simulated predictions.

## 5. Test Chip and Preliminary Results

**Figure 10:**
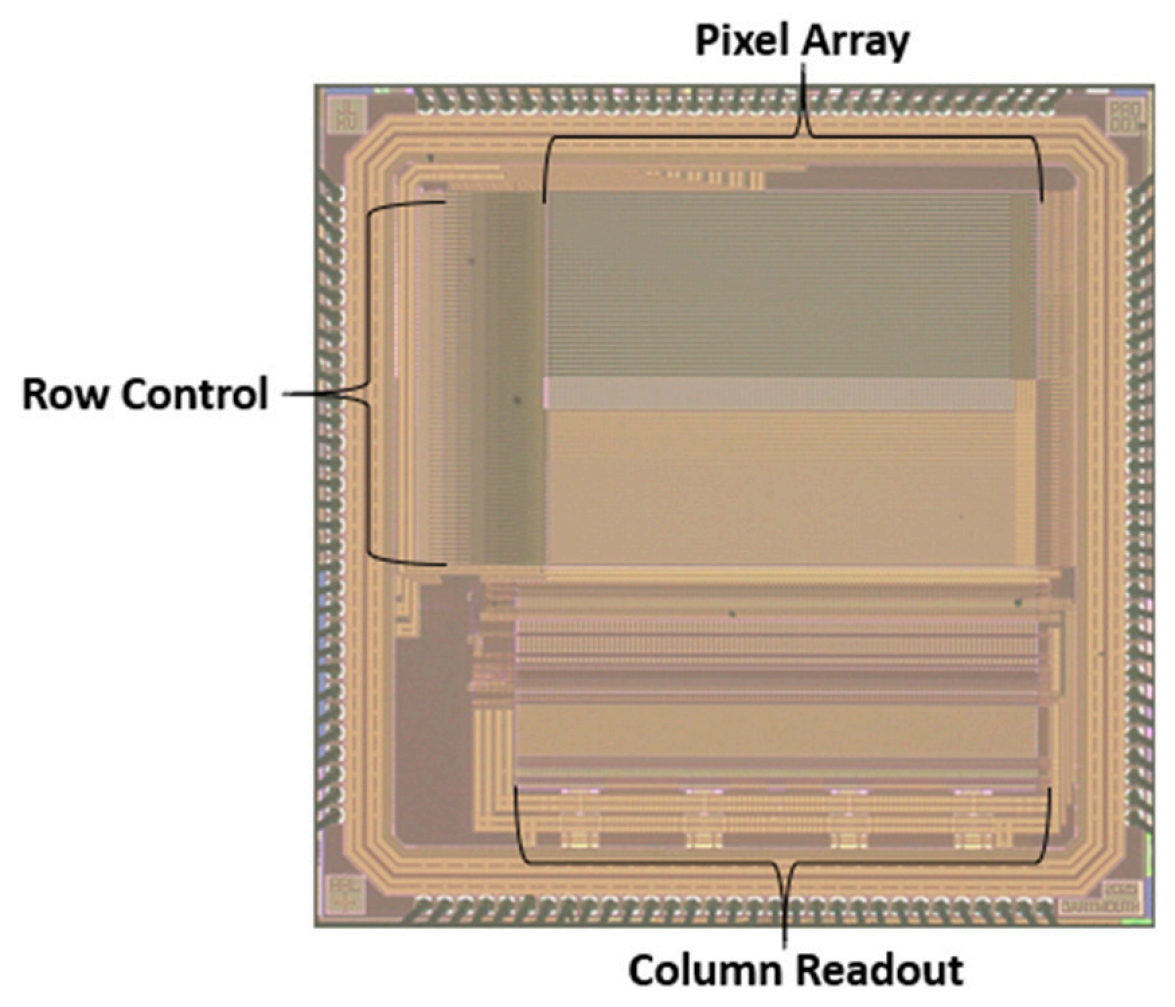
Micrograph of the wire-bonded PRO pixel test chip fabricated in X-FAB’s XS018 process.

A 10 mm^2^ test chip with a PRO pixel array was designed in a 180 nm process with the primary objective of demonstrating the programmability feature of this pixel architecture as well as to determine an optimal readout amplifier design for low-light imaging. Future PRO pixel designs will include more readout amplifiers to achieve all of the possible readouts discussed in Section 2 based on the characterization results of this test design. A micrograph of the fabricated chip is presented in Figure 10. The row control shown in this figure provides the pixel controls that bias the PRO Pixel gates as well as a row decoder for addressing the pixel rows. The column readout provides SF bias currents, programmable gain amplifiers, sample and hold circuits, and multiplexers that output to off-chip analog-to-digital converters. The analog signal output from the test chip has separate lines for signal and reset. Hence, analog or digital CDS has to be implemented off-chip. The PRO Pixel array is 36 rows by 94 columns with a pixel pitch of 20 µm. Within this array exists nine different PRO pixel variants, though the core shape of the PRO pixel is the same in each. The difference in these variants and their performance was described in previous sections.

**Figure 11:**
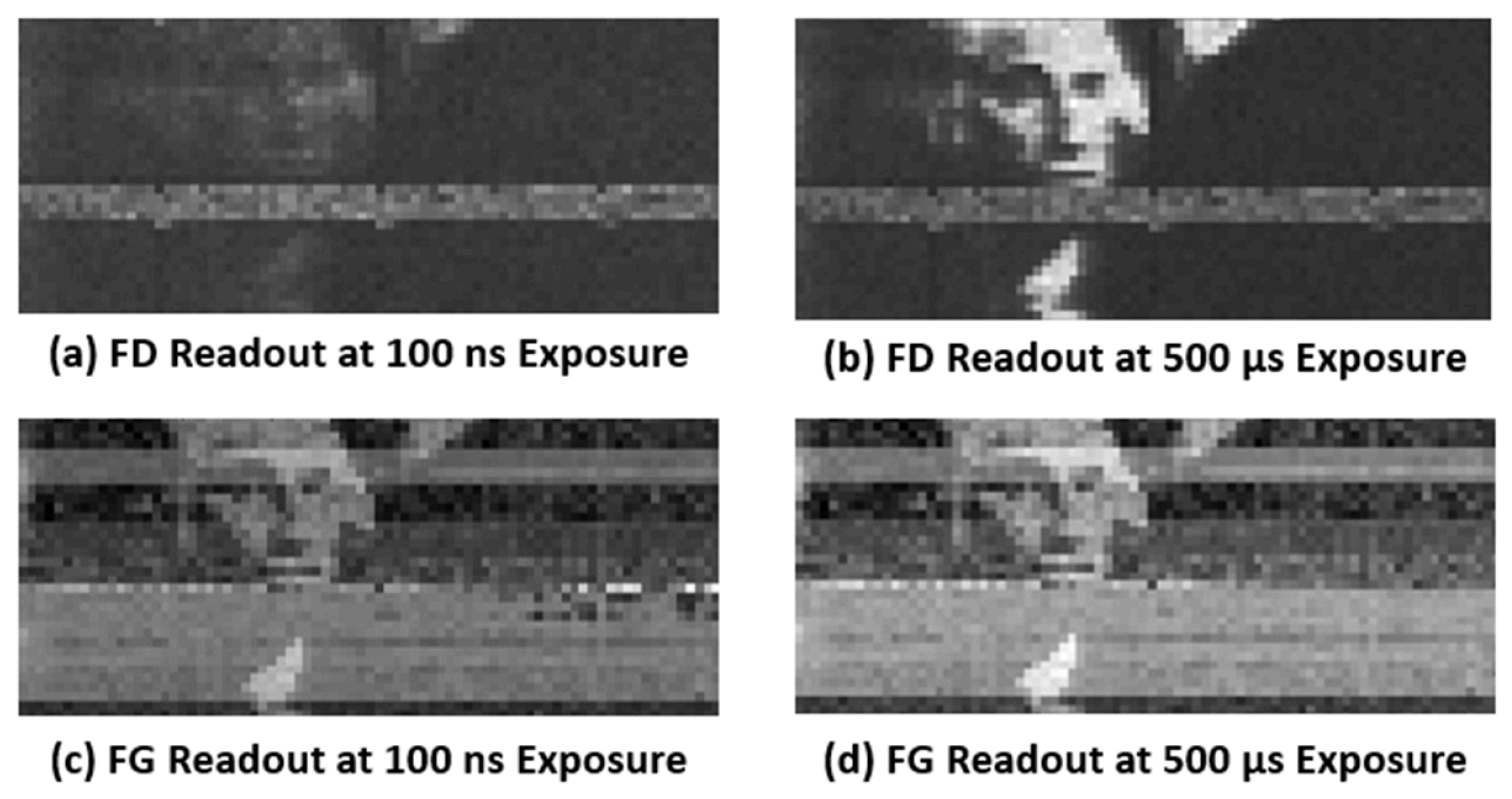
(**a**) Test image captured using a 100 ns exposure time and the floating diffusion amplifier. (**b**) Test image captured using a 500 µs exposure time and the floating diffusion amplifier. (**c**) Test image captured using a 100 ns exposure time and the floating gate amplifier. (**d**) Test image captured using a 500 µs exposure time and the floating gate amplifier. It is noted that the horizontal bands are due to different pixel designs on those rows.

**Figure 12:**
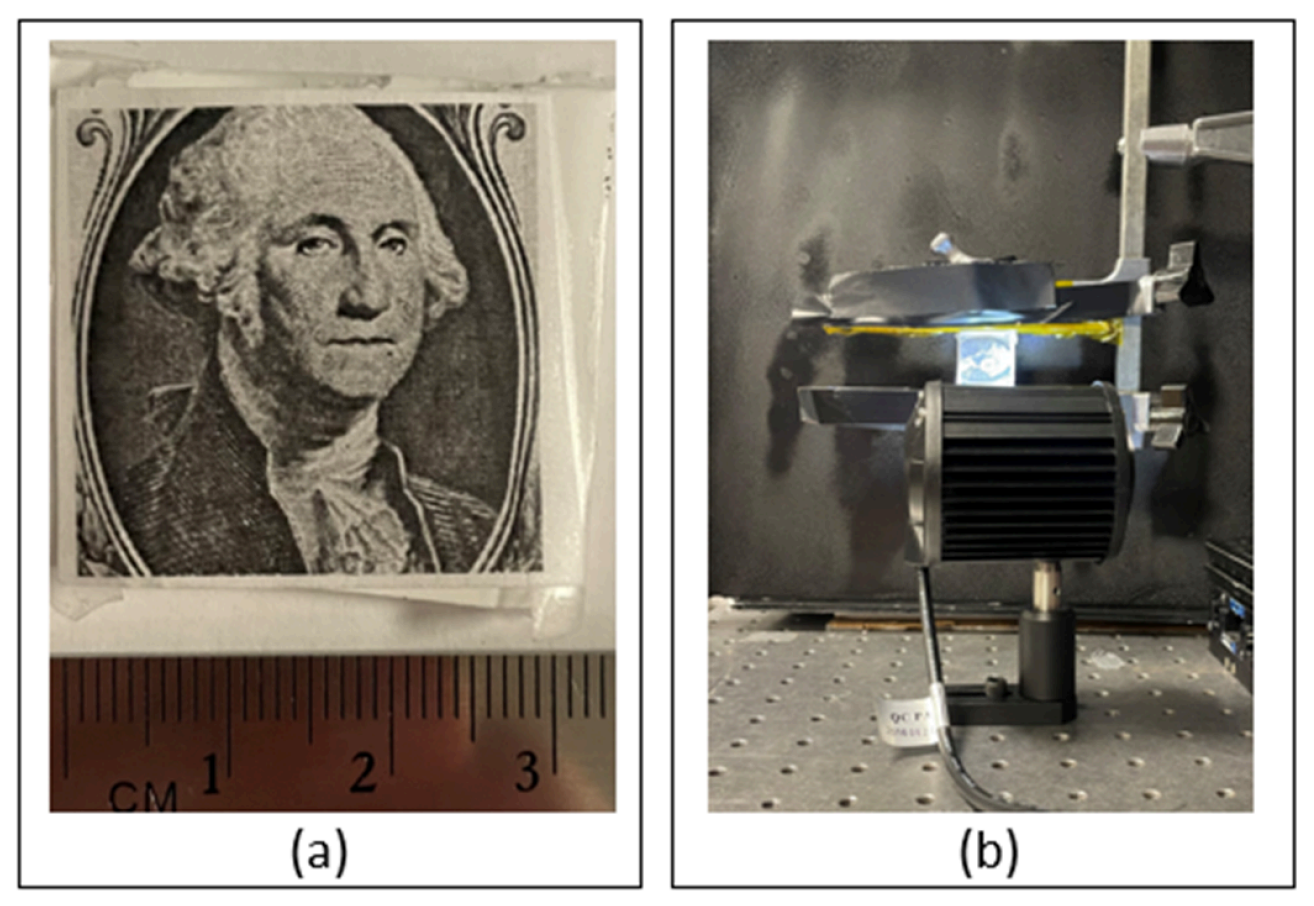
(**a**) The test image used for Figure 11 captured with a 12-megapixel iPhone 12 camera. (**b**) The imaging setup from the position where the test sensor is located.

A thorough characterization of the test chip is in progress. Preliminary experimental results show that a captured charge packet can be transported to the selected readout as demonstrated in Figure 11. Here, an image of George Washington, printed on a 3 cm × 3 cm piece of paper placed 43.5 cm from the sensor, was captured under the same lighting conditions using both the floating diffusion readout amplifier and the lower FWC floating gate amplifier at different exposure times. The floating gate amplifier was operated as a Skipper-in-CMOS by averaging 50 non-destructive samples. Figure 12 shows the original test image of George Washington captured with an iPhone 12 camera as well as the measurement setup from the sensor point-of-view, where the test image is illuminated at 6400 lux with 14 lux of reflected light reaching the test sensor.

While further tuning is required to remove unwanted artifacts in the captured image, this early visual comparison highlights the different output characteristics of the two amplifiers where the floating gate readout is shown to have higher sensitivity. This result is significant in validating the programmability feature of this test chip as the same scene is shown to be captured two different ways by only adjusting the clocking using a custom graphical user interface (GUI). By selecting the floating diffusion readout on this GUI, collected charge is routed through the charge-sweep transfer gates to the FDA (Figure 11a,b) by implementing the timing in Figure 3. If Skipper-in-CMOS readout is selected instead, the collected charge is routed through the CCD to the FGA with the clocking shown in Figure 4, and the selected number of non-destructive samples is captured and averaged (Figure 11c,d).

**Figure 13:**
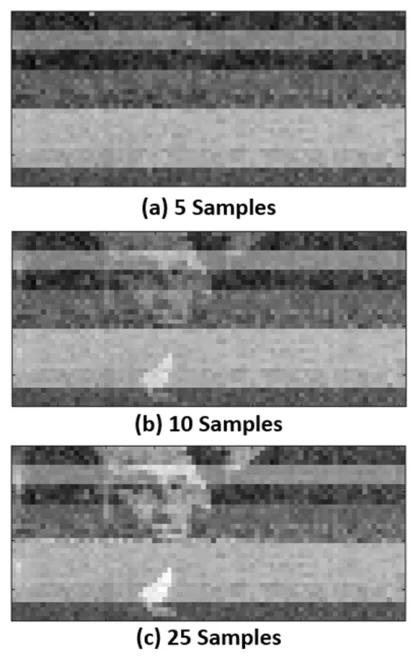
(**a**) Test image after 5 Skipper-in-CMOS samples with a 100 ns exposure time. (**b**) Test image after 10 Skipper-in-CMOS samples with a 100 ns exposure time. (**c**) Test image after 25 Skipper-in-CMOS samples with a 100 ns exposure time.

While using the floating diffusion readout, the aforementioned experimental PPD, which spans four rows, is observed to produce a horizontal stripe in the figure attributed to its unique structure that requires further study. The nine experimental source followers in the floating gate readout each have a different conversion gain responsible for the visible shading throughout Figure 11c,d. Early results demonstrate the noise reduction capability of the Skipper-in-CMOS readout as shown in Figure 13 where the image of George Washington, captured under low exposure, becomes more visible after increasing the number of samples programmed in the Skipper-in-CMOS from five in (a) to 25 in (c).

## 6. Conclusions

To achieve several desired image sensor specifications, such as low read noise, high frame rate, or high dynamic range, without requiring multiple different image sensors, a programmable readout pixel was introduced. The general concept of this PRO pixel was described where users can choose different readout methods by applying specific bias voltage sequences to CCD-in-CMOS devices to steer charge to a readout amplifier with the desired performance for a given application. Simulation data was presented which shows the expected operation of the CCD-in-CMOS and Skipper-in-CMOS devices that are integral to the functionality of a PRO pixel. A PRO pixel test chip was designed and fabricated and initial experimental results highlight that collected charge is transferred to the selected readout as expected. The test chip will be fully characterized and reported upon completion.

## Figures and Tables

**Table 1:** Test chip source follower experiments.

Variant	Transistor Type	Modulation Gate (MG) Length (µm)	Guard Gate (GG) Length (µm)	Width (µm)
1	NMOS	0.35	N/A	0.22
2	NMOS	0.65	N/A	0.22
3	NMOS	0.80	N/A	0.22
4	BC NMOS	0.65	N/A	0.22
5	MGSF	0.35	0.60	0.22
6	MGSF	0.35	1.00	0.22
7	BC MGSF	0.65	0.60	0.22
8	BC MGSF	0.65	1.00	0.22

**Table 2:** Voltages used in simulation.

Electrode	V_LOW_	V_MID1_	V_MID2_	V_HIGH_
DG	−2.0	-	-	3.3
TG	−2.0	-	-	1.5
CCD1	−2.0	0	2.0	3.3
CCD2	−2.0	0	2.0	3.3
CCD3	−2.0	0	2.0	3.3
CCD4	−2.0	0	2.0	3.3
Substrate	0.0	-	-	-
Charge Drain	-	-	-	3.3

**Table 3:** Simulated pixel readout noise for increasing source follower length.

SF Length (µm)	1/f Noise (µV rms)	Thermal Noise (µV rms)	Sense Node Cap (fF)	Conversion Gain (µV/e^−^)	Noise of First Read (e^−^)	Noise After 50 Samples (e^−^)
0.35	935	33	0.997	160	6.65	0.94
0.50	561	35	1.034	155	3.89	0.55
0.65	409	36	1.065	150	2.90	0.41
0.80	326	37	1.093	146	2.39	0.34
0.95	273	38	1.126	142	2.08	0.29

## Data Availability

The raw data supporting the conclusions of this article will be made available by the authors on request.

## Abbreviations

The following abbreviations are used in this manuscript:

DSERN	Deep sub-electron read noise
FDA	Floating diffusion amplifier
FGA	Floating gate amplifier
CCD	Charge-coupled device
CDS	Correlated double sampling
CMS	Correlated multiple sampling
GUI	Graphical user interface
SNR	Signal-to-noise ratio
JPL	Jet Propulsion Laboratory (NASA)
LOFIC	Lateral overflow integration capacitor
FWC	Full well capacity
PRO	Programmable readout
PIC	Photonic integrated circuit
